# A pilot clinical trial of recombinant human angiotensin-converting enzyme 2 in acute respiratory distress syndrome

**DOI:** 10.1186/s13054-017-1823-x

**Published:** 2017-09-07

**Authors:** Akram Khan, Cody Benthin, Brian Zeno, Timothy E. Albertson, John Boyd, Jason D. Christie, Richard Hall, Germain Poirier, Juan J. Ronco, Mark Tidswell, Kelly Hardes, William M. Powley, Tracey J. Wright, Sarah K. Siederer, David A. Fairman, David A. Lipson, Andrew I. Bayliffe, Aili L. Lazaar

**Affiliations:** 1 Div. of Pulmonary & Critical Care Medicine, Department of Medicine, Oregon Health & Science University, Portland, OR USA; 2 Riverside Methodist Hospital, Columbus, OH USA; 3 School of Medicine, University of California, Davis, Sacramento, CA USA; 4 St. Paul’s Hospital, Vancouver, BC Canada; 5 Division of Pulmonary, Allergy, and Critical Care Medicine, University of Pennsylvania School of Medicine, Philadelphia, PA USA; 6 Nova Scotia Health Authority and Dalhousie University, Halifax, NS Canada; 7 Charles LeMoyne Hospital, Sherbrooke University, Greenfield Park, QC Canada; 8 Critical Care Medicine, Vancouver General Hospital, University of British Columbia, Vancouver, BC Canada; 9 Division of Pulmonary and Critical Care, Department of Medicine, Baystate Medical Center, Springfield, MA USA; 10 GlaxoSmithKline R&D, Stockley Park, UK; 11 GlaxoSmithKline R&D, Stevenage, UK; 12 GlaxoSmithKline R&D, King of Prussia, PA USA

**Keywords:** Angiotensin-converting enzyme 2, Acute lung injury, Respiratory distress syndrome, Adult, Acute respiratory failure, Renin-angiotensin system, Humans, Interleukin-6

## Abstract

**Background:**

Renin-angiotensin system (RAS) signaling and angiotensin-converting enzyme 2 (ACE2) have been implicated in the pathogenesis of acute respiratory distress syndrome (ARDS). We postulated that repleting ACE2 using GSK2586881, a recombinant form of human angiotensin-converting enzyme 2 (rhACE2), could attenuate acute lung injury.

**Methods:**

We conducted a two-part phase II trial comprising an open-label intrapatient dose escalation and a randomized, double-blind, placebo-controlled phase in ten intensive care units in North America. Patients were between the ages of 18 and 80 years, had an American-European Consensus Criteria consensus diagnosis of ARDS, and had been mechanically ventilated for less than 72 h. In part A, open-label GSK2586881 was administered at doses from 0.1 mg/kg to 0.8 mg/kg to assess safety, pharmacokinetics, and pharmacodynamics. Following review of data from part A, a randomized, double-blind, placebo-controlled investigation of twice-daily doses of GSK2586881 (0.4 mg/kg) for 3 days was conducted (part B). Biomarkers, physiological assessments, and clinical endpoints were collected over the dosing period and during follow-up.

**Results:**

Dose escalation in part A was well-tolerated without clinically significant hemodynamic changes. Part B was terminated after 39 of the planned 60 patients following a planned futility analysis. Angiotensin II levels decreased rapidly following infusion of GSK2586881, whereas angiotensin-(1–7) and angiotensin-(1–5) levels increased and remained elevated for 48 h. Surfactant protein D concentrations were increased, whereas there was a trend for a decrease in interleukin-6 concentrations in rhACE2-treated subjects compared with placebo. No significant differences were noted in ratio of partial pressure of arterial oxygen to fraction of inspired oxygen, oxygenation index, or Sequential Organ Failure Assessment score.

**Conclusions:**

GSK2586881 was well-tolerated in patients with ARDS, and the rapid modulation of RAS peptides suggests target engagement, although the study was not powered to detect changes in acute physiology or clinical outcomes.

**Trial registration:**

ClinicalTrials.gov, NCT01597635. Registered on 26 January 2012.

**Electronic supplementary material:**

The online version of this article (doi:10.1186/s13054-017-1823-x) contains supplementary material, which is available to authorized users.

## Background

The renin-angiotensin system (RAS) regulates vascular tone and fluid-electrolyte homeostasis in a wide range of tissues [1–4]. Angiotensin II (Ang II), formed by the activity of angiotensin-converting enzyme (ACE) on angiotensin I (Ang I), is the key effector peptide of the RAS and, via the angiotensin type I receptor (AT1R), mediates physiological effects, including vasoconstriction, inflammation, apoptosis, capillary leak, and fibroproliferation [5–8]. ACE2 is a membrane-bound carboxypeptidase that hydrolyzes Ang II to the heptapeptide Angiotensin-(1–7) (Ang 1–7). ACE2 regulates RAS signaling, both directly by reducing Ang II/AT1R signaling and indirectly by activating the counterregulatory Ang 1–7/Mas receptor pathway [9–12].

RAS signaling and ACE2 have been implicated in the pathogenesis of acute respiratory distress syndrome (ARDS). Mice deficient in ACE2 developed severe acute lung injury (ALI) following challenge with a variety of insults [13, 14], which improved on repletion with recombinant ACE2 [15]. The importance of ACE/Ang II signaling in human disease is suggested by increased levels of ACE and Ang II in patients with ARDS and patients with sepsis [16–19], and it is further underlined by genetic studies of an insertion/deletion (I/D) polymorphism within the *ACE* gene, with the D allele conferring higher ACE and Ang II levels in tissue and serum [20]. A number of studies and meta-analyses [20–23] suggest an association between the *ACE* D allele and mortality in ARDS cohorts.

A recombinant version of the catalytic ectodomain of human ACE2 (rhACE2, GSK2586881) attenuated arterial hypoxemia in a piglet model of lipopolysaccharide-induced ALI [24] and was well-tolerated when administered to healthy human volunteers [25]. We postulated that the addition of exogenous ACE2 in patients with ARDS could attenuate lung injury without compromising systemic hemodynamics. We report the results of a prospective, placebo-controlled trial of GSK2586881 in mechanically ventilated patients with ARDS. The aim of the trial was to establish preliminary safety, pharmacokinetics (PK), and pharmacodynamics (PD) in critically ill patients and to explore the effects of GSK2586881 on relevant physiological measures of ARDS.

## Methods

### Study design and data collection

Between September 2012 and October 2014, we conducted a phase II study in ten intensive care units in the United States and Canada (GSK protocol ACE114622, ClinicalTrials.gov identifier NCT01597635). After institutional review board approval was obtained at each institution, written informed consent was obtained from each patient or the patient’s legally authorized surrogate prior to conduct of study-specific procedures. The study was conducted in accordance with International Conference on Harmonisation of Technical Requirements for Registration of Pharmaceuticals for Human Use Good Clinical Practice and all applicable subject privacy requirements, as well as the ethical principles outlined in the 2013 Declaration of Helsinki [26].

The study was designed in two parts. Part A was an open-label, within-subject dose escalation of GSK2586881 in hemodynamically stable patients with ARDS. The primary objective of part A was to ascertain whether ACE2 would adversely impact systemic hemodynamics in critically ill patients. Four consecutive intravenous (IV) doses of GSK2586881 (0.1 mg/kg, 0.2 mg/kg, 0.4 mg/kg, and 0.8 mg/kg) were administered to each subject at baseline and intervals of 2, 4, and 18 h, respectively. Hemodynamic assessments were carried out after each infusion and prior to escalating to the next higher dose. The 0.1 mg/kg was chosen as the starting dose because it was anticipated that this dose would produce a minimal pharmacological effect, based on the limited preclinical data derived from the piglet model and from the first-in-human study. On the basis of modelling predictions and using a conservative total dose of 0.7 mg/kg on the first day, this dose escalation strategy was not expected to result in drug accumulation. The highest dose (0.8 mg/kg) to be administered on day 2 of part A was selected to provide generous safety margins that were based on preclinical findings, because it was believed that doses < 0.8 mg/kg would prove efficacious in lowering Ang II levels.

Part B was a double-blind (sponsor unblinded) investigation comparing 3 days of twice-daily infusions of 0.4 mg/kg GSK2586881 with matched placebo. The sponsor was unblinded to allow for in-stream analysis of safety data only. The exploratory statistical decision-making framework related to PD, physiological, and clinical endpoints, as well as all outputs and reporting and analysis plans, were all defined, prepared, and approved prior to unblinding of any part B data. Subjects were randomized using a 1:1 allocation. Dose selection was based on modeling of PK and PD profiles (e.g., Ang II levels) of healthy subjects dosed with IV GSK2586881 in previous trials [25] and dose-response/duration relationships established in large animal ARDS models [24]. The primary objective of part B was to assess the safety and tolerability of GSK2256881, including adverse event (AE) reporting, clinical laboratory tests and immunogenicity, vital signs, electrocardiograms, and physical examinations. Secondary endpoints included an assessment of PK, PD, and biomarkers (details described in Additional file 1). Physiological and clinical endpoints were considered exploratory. On the basis of the anticipated pharmacology of the compound, 3 days of dosing was felt to be adequate to define the initial safety of the drug in critically ill patients, its PK, and to show a PD effect.

### Inclusion and exclusion criteria

Eligible patients included male or female subjects 18–80 years of age who were diagnosed with ARDS within 48 h of randomization that was associated with infection, sepsis, pneumonia, aspiration, or similar disease, based on the American-European Consensus Criteria [27]. Subjects were enrolled if hemodynamically stable in the 4–6 h preceding the initiation of study treatment, with stable pressor requirements, on mechanical ventilation for < 72 h before dosing began, and were managed with low tidal volume mechanical ventilation. Full eligibility criteria are described in Additional file 1.

### Statistical methods

Part B of the study was designed to randomize 60 subjects, with planned interim analyses after approximately half the subjects had completed 7 days of follow-up. In the original protocol, the interim analysis in part B was planned to allow for a sample size reestimation based on Ang II and Ang 1–5 responses. Following a planned review of data from five subjects in part A in which the effects of GSK2586881 on RAS peptides were clearer than expected, the protocol was amended to switch the objective of the part B interim analysis from confirmation of pharmacology (e.g., plasma Ang II and Ang 1–5) to a futility analysis assessing the impact on ratio of partial pressure of arterial oxygen to fraction of inspired oxygen (PaO_2_/FiO_2_) as a surrogate for potential beneficial physiological activity. This change was implemented prior to reviewing/unblinding of any data from part B.

A Bayesian statistical framework was employed, which allows quantitative statements of statistical significance to be constructed from posterior distributions [28]. This approach was considered most appropriate for a study where the potential treatment effects of the investigational medicine were less well defined. Additional details are provided in Additional file 1.

## Results

**Fig. 1:**
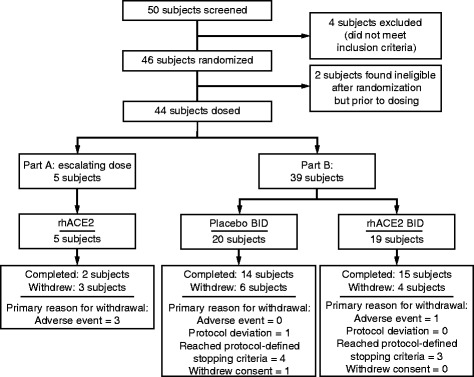
(Consolidated Standards of Reporting Trials (CONSORT) diagram of subject disposition

Forty-six subjects were enrolled, of whom 44 (5 in part A and 39 in part B) received at least one dose of study medication (Fig. 1). In part B, 16 of 20 patients on placebo and 16 of 19 patients receiving GSK2586881 received all 6 planned doses. Baseline characteristics and demographics are shown in Table 1 and Additional file 1: Table S1. At baseline, subjects who received GSK2586881 in part B had higher Sequential Organ Failure Assessment (SOFA) scores and lower PaO_2_/FiO_2_ ratios than patients receiving placebo (Table 1); other characteristics were similar between groups. The study was terminated after randomizing 39 of the planned 60 patients in part B, following the planned futility analysis.

**Table 1:** Demographic and baseline characteristics

	Part A	Part B	
	rhACE2 0.1 -> 0.2 -> 0.4 -> 0.8 mg/kg	Placebo BID	rhACE2 0.4 mg/kg BID
Number of subjects planned	5	30	30
All subjects population	5	20	19
Age, years, mean (SD)	50.8 (17.04)	50.5(15.44)	50.6(16.36)
Sex, *n* (%)			
Female	2 (40)	7 (35)	6 (32)
Male	3 (60)	13 (65)	13 (68)
BMI, kg/m^2^, mean (SD)	31.4 (5.43)	29.59 (6.896)	29.21 (4.997)
Time since ARDS, h	17.8 (10.8)	26.899 (13.82)	26.916 (13.99)
Glasgow Coma Scale	6.6 (2.51)	8.2 (4.47)	7.1 (3.20)
SOFA score	10.8 (2.49)	7.8 (2.79)	8.9 (2.36)
PaO_2_/FiO_2_, geometric mean (SD on logarithmic scale)	140.3 (0.468)	160.5 (0.523)	143.6 (0.522)
PEEP, cmH_2_O	14.0 (0.196)	10.4 (0.438)	10.4 (0.340)
Ang II, pg/ml	26.5 (0.513)	11.4 (1.834)	19.6 (1.858)

*Abbreviations: Ang* Angiotensin, *ARDS* Acute respiratory distress syndrome, *BMI* Body mass index, *PaO*_*2*_ */FiO*_*2*_ Ratio of partial pressure of arterial oxygen to fraction of inspired oxygen, *PEEP* Positive end-expiratory pressure, *rhACE2* Recombinant human angiotensin-converting enzyme 2, *SOFA* Sequential Organ Failure Assessment

### Safety and tolerability

In part A, no clinically significant changes in hemodynamic parameters were observed. The most commonly reported AE was atrial fibrillation (Additional file 1: Table S2); no AEs were considered drug-related. Three subjects who died had ten serious adverse events (SAEs). None of the SAEs was considered drug-related, with the exception of one subject who developed acute renal failure 4 days after the last dose of study drug (Additional file 1: Table S5).

In part B, 29 (75%) subjects experienced an AE (Additional file 1: Table S3). Hypernatremia, rash, dysphagia, and pneumonia occurred more frequently in subjects receiving GSK2586881. Three subjects in each treatment group had AEs that were considered by the investigator to be possibly related to study drug (Additional file 1: Table S4). Four (20%) subjects on placebo experienced six SAEs, and three (16%) subjects treated with GSK2586881 experienced four SAEs (Additional file 1: Table S5). Three subjects experienced fatal AEs, two in the placebo group (multiorgan failure and septic shock) and one in the GSK2586881 group (anastomotic dehiscence following lobectomy). None of the fatal events was considered related to study treatment. One patient on placebo was withdrawn because of increased hepatic transaminases. There were ten deaths in part B, including six (30%) subjects on placebo and four (21%) subjects who received GSK2586881. A full listing of AEs is included in Additional file 1: Tables S2–S5.

### Pharmacodynamics and biomarkers

#### RAS biomarkers

**Fig. 2:**
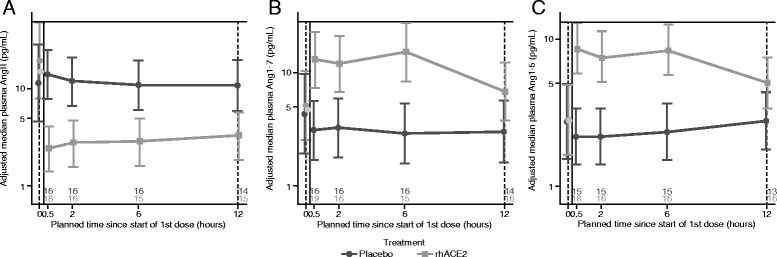
Change from baseline in plasma concentrations of angiotensin II (Ang II) (**a**), Ang 1–7 (**b**), and Ang 1–5 (**c**) following treatment with placebo or GSK2586881 (recombinant human angiotensin-converting enzyme 2 [rhACE2]). Data are expressed as adjusted median ± 95% credible interval (CrI). n* is number of subjects available for each measurement

Baseline concentrations of plasma Ang II varied considerably between subjects (Additional file 1: Figure S1). In patients who received GSK2586881, Ang II levels decreased dramatically after infusion and were sustained for up to 5 days (Fig. 2a). The nadir of Ang II was observed within the first 12 h and as early as 30 minutes following dosing with GSK2586881. In contrast, Ang II levels in subjects receiving placebo remained elevated over the first 5 days and decreased thereafter. Plasma Ang 1–7 (Fig. 2b) and Ang 1–5 (Fig. 2c) levels increased rapidly and were sustained over the first 12 h following dosing with GSK2586881 and remained substantially elevated. Similarly, Ang 1–5 levels (a product of Ang 1–7 catabolism) also increased rapidly over the first 6 h and remained elevated over a time frame similar to that of Ang 1–7 (Fig. 2c). Plasma levels of Ang 1–7 and Ang 1–5 were unchanged over the same period in patients who received placebo.

Baseline plasma Ang II concentrations were higher in nonsurvivors than in survivors (Additional file 1: Figure S2a), consistent with literature reports suggesting a link between Ang II concentrations and outcome in ARDS [16, 17]. Although baseline concentrations were higher in patients with ARDS than previously reported for healthy subjects [25], overall Ang II levels were low (Table 1). Among patients in part B, 44% presented with concentrations < 10 pg/ml (within the normal range), and the majority of subjects recruited (~70%) had Ang II concentrations < 50 pg/ml (Additional file 1: Figure S2b).

Plasma renin levels were decreased in both groups at 72 h compared with baseline. Aldosterone levels were decreased in subjects receiving GSK2586881 at 72 h compared with placebo; however, the difference was not significant (data not shown).

#### Other biomarkers

**Fig. 3:**
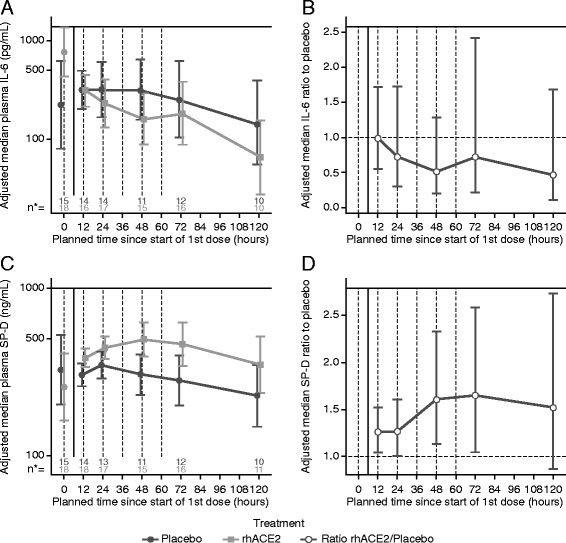
Change from baseline in plasma concentrations over time and in ratio to placebo for interleukin (IL)-6 (**a**, **b**) and surfactant protein D (SP-D) (**c**, **d**). Data are expressed as adjusted median ± 95% credible interval. n* is the number of subjects available for each measurement. *rhACE2* Recombinant human angiotensin-converting enzyme 2

Baseline serum interleukin (IL)-6 concentrations were substantially higher at baseline in the rhACE2 arm (763.6 pg/ml, 95% credible interval (CrI) 427.4–1364.4; vs 223.5 pg/ml, 95% CrI 80.1–623.6) (Fig. 3a). Following adjustment for baseline differences, there was an apparent treatment-related decrease in IL-6 concentrations in GSK2586881-treated patients compared with placebo after 24 h that did not reach statistical significance (posterior probability distribution of 0.5130–0.9254). The trend for lower IL-6 concentrations is supported by posterior probabilities of 0.92 and 0.88 that GSK2586881 reduced IL-6 levels at 48 h and 120 h, respectively (Fig. 3b).

Concentrations of surfactant protein D (SP-D) increased compared with baseline (257.7 ng/ml, 95% CrI 162.2–409.5) in subjects receiving GSK2586881, reaching a maximum at 48 h (494.7 ng/ml, 95% CrI 391.6–628.3) and then gradually decreasing (Fig. 3c). SP-D levels were significantly elevated (posterior probability > 0.95) in GSK2586881-treated subjects following dosing compared with placebo-treated subjects at 12, 24, 48, and 72 h (Fig. 3d), indicating strong evidence for a treatment-related effect.

In both groups, myeloperoxidase levels remained relatively constant for 48 h before decreasing, and there was a trend toward lower levels in placebo-treated subjects (posterior probabilities 0.93 at 24 h and 0.92 at 72 h). No significant difference was observed among treatment groups for other biomarkers, including C-X-C motif chemokine ligand 8, soluble tumor necrosis factor receptor 1, C-reactive protein, receptor for advanced glycation endproducts, Club cell protein-16, angiopoietin-2, von Willebrand factor, or plasminogen activator inhibitor 1. Tumor necrosis factor-α concentrations were below the level of assay quantification (23.5 pg/ml) for all samples in the GSK2586881-treated group.

### Clinical efficacy

#### Physiological and ventilatory endpoints

**Fig. 4:**
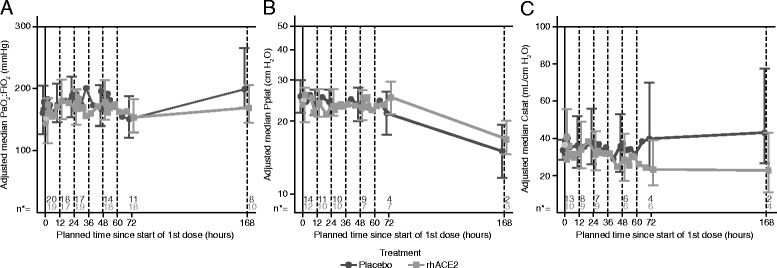
Change from baseline in ratio of partial pressure of arterial oxygen to fraction of inspired oxygen (PaO_2_/FiO_2_, **a**), plateau pressure (Pplat, **b**), and static compliance (Cstat, **c**). Data are expressed as adjusted median ± 95% credible interval. n* is the number of subjects available for each measurement

There were no significant differences in PaO_2_/FiO_2_ over the dosing period in part B, and PaO_2_/FiO_2_ was not increased at the final time point (168 h postdose) in the rhACE2 group compared with placebo (rhACE2/placebo ratio 0.85, 95% CrI 0.62–1.1) (Fig. 4a). Similarly, there were no differences between treatment groups in oxygenation index or positive end-expiratory pressure [29]. The lack of effect on oxygenation was observed regardless of baseline Ang II levels (data not shown). There were no significant differences in either peak or plateau pressures between placebo and GSK2586881 over the 72 h of treatment; however, increases in both peak and plateau pressures in those subjects who had received GSK2586881 was evident following the end of treatment. The increase in peak pressures at 72 h was statistically significant (ratio 1.28, 95% CrI 1.059–1.514, posterior probability [ratio > 1] 0.9946) (Fig. 4b). Static compliance was lower in the GSK2586881-treated group than in the placebo group throughout the study, but it decreased more notably after 60 h (Fig. 4c). The ratio for static compliance at 72 h was 0.58 (95% CrI 0.303–1.308) with a posterior probability (ratio ≥ 1) of 0.0750, suggesting a statistically significant difference favoring placebo. It should be noted, however, that the analysis at 72 h for these parameters was based on 10 subjects (4 in placebo, 6 on GSK2586881) compared with 23 at baseline (13 and 10, respectively).

#### Organ failure

SOFA scores were higher in the GSK2586881-treated group at baseline, and when they could be computed, they were also higher at all postdose time points (Additional file 1: Table S6). Notably, there was a large proportion of missing data at day 7 because of clinical improvement, which limits the conclusions that can be drawn. Fluid balance was also assessed in patients at baseline and throughout the treatment period, with no significant differences noted between treatment groups across time points (data not shown).

## Discussion

The primary objective of this study was to assess the safety of GSK2586881 in patients with ARDS, and the study also included measurements of inflammatory biomarkers and exploratory endpoints relating to lung physiology and clinical efficacy. The study met its primary endpoint because there were no episodes of hypotension associated with infusion of GSK2586881. Most AEs were equally distributed between the treatment and placebo groups and were consistent with a critically ill population; however, some were reported more frequently in subjects receiving GSK2586881, including hypernatremia; pneumonia; dysphagia; and, in particular, rash. The occurrence of rash in patients taking ACE inhibitors has been reported [30]. Rash sometimes accompanies infusions of therapeutic proteins as a result of formation of protein-protein or antibody-protein complexes that precipitate type II or III hypersensitivity reactions [30]. Although no antibody responses to GSK2586881 were detected, given the small number of subjects in this trial, the possibility of immune-mediated rash cannot be ruled out. Although all pneumonia events occurred in the treatment arm, these occurred well after the last dose of study drug (ranging from 5 to 36 days), so a clear role for GSK2586881 in the increased reports of pneumonia is difficult to establish.

Despite the increased illness severity and dysregulated RAS signaling in the GSK2586881-treated group at baseline, infusion of GSK2586881 modulated RAS peptides as expected, resulting in a significant decrease in concentrations of Ang II, accompanied by similarly rapid increases in Ang 1–7 and Ang 1–5 concentrations. This is consistent with the PK data that suggested a good correlation between plasma concentrations of GSK2256881 and measured ACE2 activity (Additional file 1: Figure S4). Whereas infusion of GSK2586881 resulted in a mean decrease in Ang II, levels in some subjects remained higher than those reported in healthy volunteers [25]. Increases in Ang 1–7 and Ang 1–5 peptide products were limited to the initial 30–60 minutes after infusion, perhaps reflecting high turnover of the initial Ang II substrate pool in the presence of high concentrations of rhACE2. This raises the possibility that continuous infusions of GSK2586881 that achieve lower plasma concentrations over a longer duration may be more effective as a result of more sustained production of Ang 1–7. Further dose regimen finding studies are required to explore these PK/PD relationships.

It is notable that 71% of patients had baseline concentrations of Ang II < 50 pg/ml, a level suggested to be of prognostic significance in some patient populations [17, 31, 32]. This observation highlights the variability in RAS activation within heterogeneous cohorts of patients with ARDS and raises the possibility that RAS activation may be driving disease in only a subgroup of patients. Researchers in future studies could consider evaluating GSK2586881 only in patients with elevated Ang II and, in light of findings in animals, could further explore the impact of RAS modulation on pulmonary hemodynamics and markers of pulmonary vascular injury.

Treatment with GSK2586881 resulted in a reduction in IL-6 concentrations, although this did not reach statistical significance, owing to intersubject variability and baseline imbalances. The elevations in SP-D were unexpected and raise a number of questions about GSK2586881’s mechanism of action. SP-D is a large collectin family protein, with expression usually restricted to the lung [33, 34]. Its presence in serum has been suggested to be an indicator of worsening alveolar capillary permeability. However, SP-D is also an anti-inflammatory [33, 34] and antimicrobial protein [35]; thus, the observed increases could be reflective of increased SP-D biosynthesis in the lung as a result of GSK2586881 treatment. These data highlight a need for further research on the potential mechanistic link between ACE2 and SP-D biology.

Although difficult to assess because of study limitations, it is possible that treatment with GSK2586881 worsened respiratory mechanics, with the change in compliance and ventilatory pressures possibly suggesting an increase in lung stiffness [29, 36, 37]. Although most of the biomarkers measured suggested no change or reduced disease activity in GSK2586881-treated subjects, the increase in myeloperoxidase is difficult to explain on the basis of known ACE2 biology and previous effects in animals and humans, and it could reflect lung neutrophil accumulation and the potential for altered respiratory mechanics. Some of the analyses were impacted by missing data (e.g., subject withdrawal, extubation, technical issues, early mortality); therefore, the number of subjects supporting these comparisons was small, significantly increasing the possibility of systematic bias at later time points. There were baseline imbalances in severity of illness (based on SOFA score and serum IL-6 and Ang II levels) and case mix between treatment groups.

The lack of improvement in oxygenation in patients receiving GSK2586881 contrasts with effects reported in large animal models of ARDS, where IV rhACE2 rapidly improved arterial hypoxemia and pulmonary hemodynamics [15, 38]. Although PaO_2_/FiO_2_ and other ventilatory parameters are important in the diagnosis of ARDS and in determining the severity of hypoxemia, they cannot be standardized clinically to the same extent as in animal studies, and they are influenced by numerous factors that were not adequately controlled for in this trial. These issues limit interpretation of the effects of GSK2586881 on oxygenation and ventilatory parameters.

## Conclusions

Infusion of GSK2586881 resulted in the expected changes in RAS biomarkers and were well-tolerated in subjects with ARDS. However, GSK2586881 infusions did not result in improvement in physiological or clinical measures of ARDS in this small study. Because the primary objective (preliminary safety, PK, and PD) was met at the interim analysis, and because statistical trial simulations of the interim data predicted with reasonable confidence that the outcome at trial completion (*n* = 60) would be similar to the interim outcome, continued recruitment was not justified, and the trial was terminated early. Further exploration of the effects of GSK2586881 in ARDS will need to be built on a better understanding of the role of RAS in ARDS pathophysiology in humans, as well as of the effects of rhACE2 on pulmonary physiology.

## Additional files

Additional file 1: Online Supplement to Pilot trial of ACE2 in ARDS. (DOCX 408 kb)

Additional file 2: Ethics Committees and Institutional Review Boards. (DOCX 13 kb)

## Abbreviations

ACE2: Angiotensin-converting enzyme 2
AE: Adverse event
ALI: Acute lung injury
Ang: Angiotensin
ARDS: Acute respiratory distress syndrome
AT1R: Angiotensin type I receptor
BMI: Body mass index
CONSORT: Consolidated Standards of Reporting Trials
CrI: Credible interval
I/D: Insertion/deletion
IL: Interleukin
IV: Intravenous
LLQ: Lower limit of quantitation
PaO_2_/FiO_2_: Ratio of partial pressure of arterial oxygen to fraction of inspired oxygen
PD: Pharmacodynamics
PEEP: Positive end-expiratory pressure
PK: Pharmacokinetics
RAS: Renin-angiotensin system
rhACE2: Recombinant human angiotensin-converting enzyme 2
SAE: Serious adverse event
SP-D: Surfactant protein D
SOFA: Sequential Organ Failure Assessment
