# Supplementary material for: A pilot clinical trial of recombinant human angiotensin-converting enzyme 2 in acute respiratory distress syndrome
Source: Crit Care. 2017 Sep 7;21:234. doi: 10.1186/s13054-017-1823-x (PMC5588692; doi:10.1186/s13054-017-1823-x)
Supplement: Supplementary file 1 — Online Supplement to Pilot trial of ACE2 in ARDS. (DOCX 408 kb) [file 13054_2017_1823_MOESM1_ESM.docx]

**Online Data Supplement (**additional details available at <http://www.gsk-clinicalstudyregister.com/files2/GSK-114622-Clinical-Study-Result-Summary.pdf>)

**A Pilot Clinical Trial of Recombinant Human Angiotensin Converting Enzyme 2 GSK2586881 in Acute Respiratory Distress Syndrome**

**Table of Contents**

Inclusion/Exclusion Criteria

Statistical Modeling

RAS peptide methods and additional results

Figure E1: Individual Ang II levels, Part B

Figure E2: Angiotensin II distribution, Part B

Safety and additional outcomes

Table E1: Additional demographics

Table E2, E3: All Adverse events, Parts A and B

Table E4: Drug-related adverse events, Part B

Table E5: Serious adverse events

Table E6: SOFA scores

Immunogenicity

Pharmacokinetics methods and additional results

Figure E3: Pharmacokinetics of GSK2586881

Figure E4: GSK2586881 Plasma Concentration vs ACE2 Enzymatic activity

**INCLUSION CRITERIA**

Eligible patients included male or female subjects, 18 - 80 years of age; diagnosed with ARDS associated with infection, sepsis, pneumonia, aspiration or similar who were hemodynamically stable (low-dose arginine vasopressin ( ≤0.04 units/min) was not considered a pressor). Patients were eligible if diagnosed with ARDS within 48 hours of randomization and ventilated for less than 72 hours. Subjects had to have a QT duration corrected for heart rate by Bazett’s formula (QTcB) or QT duration corrected for heart rate by Fridericia’s formula (QTcF) ≤ 480 msec.

At the time of writing the protocol, ARDS was defined using the AECC criteria, as a PaO2/ FiO2 ratio ≤ 300 (if altitude >1000 m then PaO2/FiO2 ratio ≤300 [X barometric pressure/760]) and bilateral infiltrates consistent with non-hydrostatic pulmonary edema on frontal chest radiograph. Patients must require positive pressure ventilation via an endotracheal tube, and have no clinical evidence of left atrial hypertension (i.e. a pulmonary capillary occlusion pressure <18 mm Hg if measured).

**EXCLUSION CRITERIA**

Subjects were excluded from the study if they were hemodynamically unstable and in the opinion of the investigator would be unable to complete the study. Subjects with positive Hepatitis B surface antigen, Hepatitis C antibody or Human Immunodeficiency Virus (HIV) antibody, current or chronic history of liver disease (Child Pugh score ≥10), or known hepatic or biliary abnormalities (with the exception of Gilbert's syndrome or asymptomatic gallstones), known history of substance abuse or alcohol abuse, within 6 months of the study causing chronic liver disease such as cirrhosis, chronic ascites or portal hypertension, or known evidence of withdrawal syndrome within the past 6 months were excluded.

Other exclusion criteria included: inability to discontinue use of Angiotensin converting enzyme type 1 inhibitors or Angiotensin receptor blockers. Subjects requiring high doses of loop diuretics (i.e. > 240 mg furosemide daily) with significant intravascular volume depletion, as assessed clinically; history of sensitivity to any of the study medications, or components there of or a history of drug or other allergy that contraindicated their participation; pregnant and lactating females; subjects with history of sensitivity to heparin or heparin-induced thrombocytopenia. Unstable Hemoglobin (Hb < 7 mg/dL) at time of drug infusion (i.e. Hb had to be ≥ 7 mg/dL at the time of drug infusion. Transfusion was permitted to increase Hb concentrations to allow entry into the study). Malignancy or other irreversible condition for which 6 month mortality was estimated to be >50%). Arterial blood pH less than 7.1 or serum bicarbonate (HCO3^-^) <15 mEq/L (if arterial blood gas [ABG] not available) before infusion was started (e.g. resuscitation to raise pH ≥ 7.1 or serum HCO3^-^ ≥ 15 mEq/L was permitted and subject could then be dosed). Known severe chronic respiratory disease with known Forced Expiratory Volume in 1 second (FEV1)/ Forced Vital Capacity (FVC) less than 45% predicted, or known chronic hypercapnia (partial pressure of carbon dioxide in arterial blood [PaCO2]> 45 millimeters of mercury [mmHg]) or chronic hypoxemia (PaO2<55 mmHg) on FiO2 =0.21, or known FEV1 <15 mL/kg (e.g. 1L for 70 kg person). Known radiographic evidence of chronic interstitial infiltration, or known hospitalization within the past six months for respiratory failure (Partial pressure of carbon dioxide in arterial blood [PaCO2]> 50 mmHg or PaO2 < 55 mmHg, or oxygen saturation <88% on FiO2 = 0.21), or known chronic restrictive, obstructive, neuromuscular, chest wall, or pulmonary vascular disease resulting in severe exercise restriction (i.e. unable to climb stairs or perform household duties). Known secondary polycythemia, severe pulmonary hypertension, or ventilator dependency. Known history of neuromuscular disease that might affect time on mechanical ventilation or impaired ability to ventilate spontaneously (such as amyotrophic lateral sclerosis, Guillain-Barre Syndrome, and myasthenia gravis). Vasculitis with diffuse alveolar hemorrhage, lung transplantation, pre-existing renal failure on hemodialysis or peritoneal dialysis requiring renal replacement therapy. If in the judgement of the Principle Investigator or study sponsor subjects’ participation might jeopardize the health of the subject or the integrity of the study. Subjects with alanine aminotransferase (ALT) > 8xULN (subjects with ALT>5-8xULN 1 included only if bilirubin <1.5xULN).

**STATISTICAL MODELING**

The structure of the statistical analyses incorporate Bayesian versions of repeated measures mixed effect models fitted to each endpoint (separate model per endpoint). Posterior distributions for the pre-specified treatment comparisons were constructed using appropriate combinations of the fitted model parameters. These posterior distributions for the (true underlying) average treatment effects have been summarized using their medians and 95% equi-tailed credible intervals. Posterior Probability (PP) statements are derived from the areas under the curve (e.g. probability of any reduction = AUC [-∞, 0]) and therefore can be interpreted as the levels of certainty. Within this manuscript Posterior Probabilities in the region of (or exceeding) 0.9 are regarded as high certainty of the observed value representing a true treatment effect.

Since non-informative priors were used for all model parameters and the treatment comparisons test for any effect relative to placebo, the set of statistical analyses in this study can be reproduced using more familiar frequentist methodology and there is a direct relationship between the posterior probabilities presented in this manuscript and a p-value. For reference a significant p-value from a frequentist two-sided test at the 5% level (p < 0.05) is equivalent to the posterior probability (PP) exceeding 0.975 (i.e. Statistical significance occurs at the α% level for a two-sided test with no multiplicity adjustment when the PP exceeds 1 – ((α/100) / 2)).

**Angiotensin Peptide data – Part B only**: Separate Bayesian repeated measures mixed effect models, with non-informative priors for all model parameters, were fitted to each angiotensin peptide analyte (each observation was natural log transformed (Ln) prior to model fitting). Fixed terms for Intercept, Centre, Ln(Baseline) by Time, Treatment, Time and Treatment by Time were constructed (Baseline as a continuous term centered prior to model fitting, all other terms were categorical effects). The repeated measures term was the subject, with a Spatial Power variance covariance matrix (distances derived using the planned times) common to both treatment arms. Appropriate combinations of the model parameters were used to construct posterior distributions for the average treatment effect and the ratio of active to placebo (at each of the planned times). Posterior probabilities of the treatment ratio showing any increase and any decrease were derived within each time.

**Measures of oxygenation and Ventilator parameters – Part B only:** A similar modeling strategy to the Angiotensin Peptide data was employed, except only posterior probabilities of any increase were obtained. Oxygen requirement (FiO2), PaO2, Tidal Volume, Minute Ventilation, Mean Airway Pressure and oxygen saturation (SaO2) via pulse oximetry were not formally modelled. Static Compliance was derived and analyzed post-hoc to support this manuscript using the same modeling strategy and approach as the other ventilation parameters.

**Other Biomarker endpoints – Part B only:** A similar modeling strategy to the angiotensin peptide data was employed, except the variance covariance matrix was unstructured and not spatial power.

**SOFA score – Part B only:** Due to missing component data at post dose time points on both treatment arms, the planned statistical modelling was not implemented. Instead summary statistics have been presented.

**Interim Analysis (Part B)**Weighted mean PaO2/FiO2 values over 0-12hours and 0-72 hours were derived for each subject using the available data (Ln transforming the individual time points prior to deriving the weighted mean); n=14:16, Placebo:Active. A multivariate normal distribution was fitted to this data (using non-informative priors), and used to simulate pairs of weighted mean values for a sufficient number of subjects to achieve an overall total of 30 per treatment arm (the 30 made up of observed and simulated data). Each of the weighted mean endpoints was then analyzed separately and a posterior distribution was obtained (comparing Active to Placebo). The posterior distributions were assessed against pre-defined criteria and rules to obtain one of three outcomes (Stop and Review, Continue or Strong Continue). The simulation step was repeated 10,000 times and the proportions of each outcome recorded. The most frequently occurring outcome formed the IA2 recommendation. Following a Stop and Review IA2 recommendation review of the other available data, using the same outputs as planned for the final analyses, resulted in a decision to stop recruitment. The time period from PaO2/FiO2 review to having collected/processed the biomarker samples to confirm the stop recruitment decision was several months and as allowed by the protocol recruitment continued into Part B; hence the final Part B sample size increased beyond the n=30 stated in the protocol for the IA2 data cut to n=39.

**Bayesian Statistical Methodology and Trial Decision Making Framework**

Although no formal statistical techniques were used to set the sample size, some of the properties of the chosen design have been assessed using crude decision grids and computer simulations, assuming that the study is not stopped at the time of interim analysis. Data from two studies (1-2) were available to GSK and used to obtain variance/covariance estimates for two day 1 change from baseline endpoints; namely P_a_O_2_ (mmHg) and P_a_O_2_/F_i_O_2_; assuming a multivariate normal distribution. Only subjects who were a close match to the proposed inclusion/exclusion criteria for this study were used to obtain the variance estimates shown below:

The row and column numbers in Table 1 represent the difference between the mean of the test item (GSK2586881) and standard of care. For example, if the test item increases P_a_O_2_ by more than 15 mmHg over standard of care in addition to increasing P_a_O_2_/F_i_O_2_ by more than 25 over standard of care there would be a strong case to continue development of GSK2586881.

Table 1 Crude decision grid for Day 1 change from baseline

| [Test item Day 1 Change from Baseline] – [Standard of care Day 1 Change from Baseline] | | P_a_O_2_/F_i_O_2_ | |
| --- | --- | --- | --- |
|  |  | <25 | ≥25 |
| P_a_O_2 (mmHg)_ | <15 | Data do not support asset | Expert judgement required |
|  | ≥15 | Expert judgement required | Data support further development of asset |

Simulations were performed assuming three “true” scenarios about the test item, scenario 1: test item is identical to standard of care, scenario 2: the true difference is the same as the decision boundaries, and scenario 3 represents a case where the test item exhibits a clear advantage. Potential sample sizes of n=15 per arm, n=30 per arm and n=60 per arm were evaluated by simulating 100,000 simulated studies per scenario and recording the percentage of studies falling into each cell of Table 1 is shown below in Table 2.

Table 2 Percentage of simulations in each decision category

|  | | **True treatment advantage over standard of care [P_a_O_2,_ P_a_O_2_/F_i_O_2]_** | | | | | |
| --- | --- | --- | --- | --- | --- | --- | --- |
|  |  | **#1. “No difference” [0,0]** | | **#2. “On boundary” [15,25]** | | **#3. “Clear difference” [30,50]** | |
|  |  | **<25** | **≥25** | **<25** | **≥25** | **<25** | **≥25** |
| **n=15 per arm** | **<15** | 77.29 | 7.09 | 37.63 | 12.10 | 8.63 | 7.15 |
|  | **≥15** | 7.07 | 8.55 | 12.01 | 38.27 | 6.95 | 77.27 |
| **n=30 per arm** | **<15** | **88.09** | 4.21 | 37.63 | 12.10 | 3.48 | 4.21 |
|  | **≥15** | 4.20 | 3.5l1 | 12.01 | 38.27 | 4.05 | **88.26** |
| **n=60 per arm** | **<15** | 96.41 | 1.41 | 37.63 | 12.10 | 0.73 | 1.39 |
|  | **≥15** | 1.44 | 0.75 | 12.01 | 38.27 | 1.41 | 96.48 |

The rows corresponding to n=30 per arm in Table 2 indicate the ability of the chosen design to distinguish a drug that doesn’t differ from standard of care from a drug that shows a clear difference. The perfect experiment would result in 100% of outcomes in the top left cell of Table 1 under scenario 1 and 100% of outcomes in the bottom right cell of Table 1 under scenario 3. Assuming similar variation between the previous studies and this study there is an 88.09% chance the observed data would not support development when there truly is no difference between test item and standard of care, and a 3.51% chance of incorrectly concluding that the data clearly support development (akin to a Type I error rate). There is just over an 8% chance of being in the “grey area”. Table 2 can be read in a similar fashion for the remaining combinations of scenario and sample size per arm. If (by coincidence) the true treatment effect is the same as the decision grid cut off values (as in simulation scenario #2) changing the sample size would not be expected to change the percentages in each cell category (in scenario #2 the percentages are only influenced by the correlation between the two endpoints).

Although the sample size of 30 per arm was based on feasibility, the above work gives some reassurance that this number should provide an acceptable level of information on which to base decisions on whether GSK2586881 is likely to be effective in future trials, although it should be noted that the only prior expectation on the treatment effect size is that it should lie somewhere within the three simulation scenarios covered above. This study should provide estimates of the treatment effect sizes which can be used to help design subsequent clinical studies.

*Protocol Amendment #4: Appendix 3* *Technical details of Interim Analysis 2*

For each subject the inividual P_a_O_2_/F_i_O_2_ ratios measured at the times described in the Time and Events Table will be used to derive weighted means over 0 to 12 hours (initial drug effect) and 0 to 72 hours (maintenance of drug effect).

A Bayesian approach will be used to obtain information from the posterior predictive distribution for the treatment effect, relative to the placebo, for each endpoint assuming the study were to run to the planned maximum (60 subjects). Table 4 shows the components that make up decision rules that will be used to assess the effect of GSK2586881 relative to Placebo. The cut-off points listed have been expressed as differences for ease of clinical interpretation and as ratios for the analysis itself (since historical data suggested a log transformation may be necessary).

Data for a derived endpoint will be declared positive, provided the PPP (posterior predicitive probability) that the treatment effect cut-off exceeds the required level of confidence. Using the first row as an example the logic becomes “declare positive data for WM P:F ratio 0-12h if PPP(Trt Ratio > 1.10) > 0.95”. Negative data would be declared if PPP(Trt Ratio > 1.00) < 0.5. Inconclusive data would be declared if there is not strong enough evidence to meet the positive or negative data confidence levels.

Table 5 describes the decisions associated with each of the nine possible outcomes used to assess the operating characteristics.

Table 4 Components of Part B Interim Analysis Decision Criteria

| **Derived Endpoint** | **Expressed as...** | **Cut-off for...** | | **Confidence level required** | |
| --- | --- | --- | --- | --- | --- |
|  |  | **Positive data** | **Negative data** | **Positive data** | **Negative data** |
| **WM P:F ratio 0-12h** | **Difference (Act – Pbo)** | +20 | 0 | 95% | 50% |
|  | **Ratio  (Act / Pbo)** | 1.10 | 1.00 |  |  |
| **WM P:F ratio 0-72h** | **Difference (Act – Pbo)** | +10 | 0 | 95% | 50% |
|  | **Ratio  (Act / Pbo)** | 1.05 | 1.00 |  |  |

Table 5 Decision grid for Part B Interim Analysis outcomes

| **Strength of Evidence** | | **WM P:F ratio 0-12h** | | |
| --- | --- | --- | --- | --- |
|  |  | **Negative data** | **Inconclusive** | **Positive data** |
| **WM P:F ratio 0-72h** | **Positive data** | Continue | Strong continue | Strong continue |
|  | **Inconclusive** | Stop and review | Continue | Strong continue |
|  | **Negative data** | Stop and review | Stop and review | Stop and review |

**Obtaining the Posterior Predictive Distribution**

For an individual subject the 2x weighted means will be assumed to follow a multivariate normal distribution. Separate mean vectors will be fitted for each treatment arm, but each treatment will share a common variance covariance matrix. Appropriate non-informative priors will be constructed for the mean vectors (each entry in the mean vectors will be assigned a normal distribution centred on zero with variance of 1E6) and the variance covariance matrix (Wishart distribution). SAS PROC MCMC will be used to combine the non-informative priors with the data observed at the interim to obtain 10,000 draws from the posterior distribution of the mean and variance covariance matrix parameters. For each draw (taken after the model has convergence) sufficient new pairs of data points will be sampled from that instance of posterior distribution to bring the overall total to 60 subjects (30 per treatment arm). Each set will be combined with the observed interim data to form 10,000 potential completed studies.

The 0-12h and 0-72h endpoints in these datasets of potential completed studies will be analysed separately. Assuming non-informative priors the posterior distribution for the treatment ratios will be obtained and used to check which of the outcomes listed in Table 4 are supported (i.e. positive, negative or inconclusive data). The criteria specified in Table 5 will be used to categorise each potential study into a Strong Continue, Continue or Stop and review outcome and the expected probability of each decision will be determined by the number of studies resulting in that decision divided by 10,000. The recommendation of the interim analysis will be the decision with the largest probability.

Timing of interim analysis 2 in Part B and expected Operating Characteristics

Operating characteristics of the methodology used in interim analysis 2 were assessed as follows.

The daily P_a_O_2_/F_i_O_2_ data up to day 7 from the two studies described in Section 6.2.2 were log transformed (natural log) and used to estimate the parameters of a spatial power variance covariance structure (sigma^2 = 0.1962, rho = 0.9831, time measured in hours). These parameters were used with the planned Part B sampling schedule (see Time and Events table) to obtain the expected variance covariance matrix of the individual Part B P_a_O_2_/F_i_O_2_ values, for an individual subject. Since, the weighted means (0-12h and 0-72h) are derived via summing appropriate multiples of each of the P_a_O_2_/F_i_O_2_ values, the variance covariance matrix of the weighted means for an individual subject may also be obtained via simple algebra. Thus, supplying expected values for the individual P_a_O_2_/F_i_O_2_ sampling times allows weighted means for an individual subject to be simulated from a bivariate normal distribution. For simplicity the placebo P_a_O_2_/F_i_O_2_ values were all assumed to be 200 and the active P_a_O_2_/F_i_O_2_ time profiles were varied to obtain operating characteristics. One thousand separate study datasets containing data up to the proposed number of subjects at the interim were generated from the bivariate normal distributions from each of 5 potential scenarios. The procedure for obtaining the interim analysis 2 decision (described above) was run on each of these 1,000 potential interim datasets and the outcome recorded. The operating characteristics reported are the proportions of the 1,000 initial sets of data resulting in each outcome. Factors that were investigated in the simulation exercises were 5x scenarios of true time profile of the active P_a_O_2_/F_i_O_2_ values (including the null case where the active profiles were all 200), number of subjects at the interim, confidence level associated with each cut-off in Table 4. The full outputs of this exercise are available on file, and the final criteria are as described in this document, and are expected to provide an acceptable level of performance under a variety of drug effects and durations (see Table 6).

Table 6 Expected operating characteristics of Part B interim 2

| **Scenario** | **Description of P_a_O_2_/F_i_O_2_ time profile**  **(Placebo P:F and 0h active values always 200)** | **Proportion of simulations resulting in a recommendation of …** | | |
| --- | --- | --- | --- | --- |
|  |  | **Strong Continue** | **Continue** | **Stop and Review** |
| Null | Active P:F all 200 | 0.108 | 0.194 | 0.698 |
| +20 P:F | Active Post 0h all 220 | 0.285 | 0.301 | 0.414 |
| +50 P:F | Active Post 0h all 250 | 0.620 | 0.240 | 0.140 |
| Tapering | Active Post 0h to 24h 250 then linear decline to 200 by 72h | 0.495 | 0.292 | 0.213 |
| +35 P:F | Active P:F all 235 | 0.443 | 0.303 | 0.254 |

**RAS PEPTIDES
Assay Methods**
For RAS peptide analysis, whole blood was collected into EDTA plasma tubes containing a proprietary cocktail of protease inhibitors to prevent ex vivo enzymatic degradation of the angiotensin peptides. Ang II, Ang 1-7 and Ang 1-5 levels in plasma were determined using a surrogate analyte strategy similar to that described for small molecule biomarkers with high performance liquid chromatography-tandem mass spectrometry (HPLC-MS/MS) detection (Jones et al. *Bioanalysis* 4:2343, 2012).

**Results**

**Figure S1.** Individual subject Ang II concentrations following treatment with placebo (A) or GSK2586881 (B).

**Figure S2**. (A) Baseline AngII concentrations in survivors and non-survivors by treatment. Boxes express intra-quartile range. Open circle or + sign-mean; solid line-median; outliers expressed as smaller open circles or + signs; dotted line-LLQ for assay. (B) Distribution of baseline AngII concentrations at study entry by treatment.

**ADDITIONAL SAFETY AND OUTCOMES**

**Table S1 Additional Demographics and Baseline Characteristics**

| **Number of Subjects** | **Part A** | **Part B** | |
| --- | --- | --- | --- |
|  | **rhACE2 0.1 -> 0.2 -> 0.4 -> 0.8 mg/kg** | **Placebo BID** | **rhACE2 0.4 mg/kg BID** |
| All Subjects | 5 | 20 | 19 |
| **Height** (cm) [Mean (SD)] | 176.6 (11.72) | 171.0(10.15) | 169.6(8.33) |
| **Weight** (kg) [Mean (SD)] | 97.8 (19.33) | 87.43(25.236) | 84.01(15.389) |
| **Ethnicity** [n (%)] | | | |
| Hispanic or Latino: | 0 | 0 | 2 (11) |
| Not Hispanic or Latino: | 5 (100) | 20(100) | 17 (89) |
| **Race** [n (%)] | | | |
| African American/African Heritage | 0 | 1 (5) | 0 |
| American Indian or Alaskan Native | 0 | 1 (5) | 0 |
| Asian – East Asian Heritage | 0 | 0 | 1 (5) |
| Asian – Japanese Heritage | 0 | 1 (5) | 0 |
| White – White/Caucasian/European Heritage | 5 (100) | 17 (85) | 18 (95) |

**Table S2 Summary of all Adverse Events, Part A**

| **System Organ Class Preferred Term** | **rhACE2 0.1 -> 0.2-> 0.4-> 0.8 mg/kg** (N=5) |
| --- | --- |
| ANY EVENT | 5 (100%) |
|  |  |
| **Respiratory, thoracic and mediastinal disorders** |  |
| Any event | 4 (80%) |
| Acute respiratory distress syndrome | 1 (20%) |
| Epistaxis | 1 (20%) |
| Pneumothorax | 1 (20%) |
| Respiratory failure | 2 (40%) |
|  |  |
| **Blood and lymphatic system disorders** |  |
| Any event | 3 (60%) |
| Anaemia | 2 (40%) |
| Disseminated intravascular coagulation | 1 (20%) |
|  |  |
| **Cardiac disorders** |  |
| Any event | 3 (60%) |
| Atrial fibrillation | 3 (60%) |
| Cardiac failure | 1 (20%) |
|  |  |
| **Gastrointestinal disorders** |  |
| Any event | 3 (60%) |
| Abdominal pain | 1 (20%) |
| Constipation | 1 (20%) |
| Diarrhoea | 2 (40%) |
| Nausea | 1 (20%) |
| Upper gastrointestinal haemorrhage | 1 (20%) |
| Vomiting | 1 (20%) |
|  |  |
| **General disorders and administration site conditions** |  |
| Any event | 3 (60%) |
| Generalised oedema | 1 (20%) |
| Injection site haemorrhage | 1 (20%) |
| Multi-organ failure | 1 (20%) |
| Pyrexia | 1 (20%) |
|  |  |
| **Infections and infestations** |  |
| Any event | 2 (40%) |
| Influenza | 1 (20%) |
| Sepsis | 1 (20%) |
|  |  |
| **Investigations** |  |
| Any event | 2 (40%) |
| Blood calcium decreased | 1 (20%) |
| Blood magnesium decreased | 1 (20%) |
| Blood phosphorus decreased | 1 (20%) |
| Blood sodium increased | 1 (20%) |
| Body temperature increased | 1 (20%) |
| Oxygen saturation decreased | 1 (20%) |
|  |  |
| **Metabolism and nutrition disorders** |  |
| Any event | 2 (40%) |
| Acidosis | 1 (20%) |
| Hyperglycaemia | 1 (20%) |
|  |  |
| **Nervous system disorders** |  |
| Any event | 2 (40%) |
| Headache | 1 (20%) |
| Subarachnoid haemorrhage | 1 (20%) |
|  |  |
| **Psychiatric disorders** |  |
| Any event | 2 (40%) |
| Agitation | 1 (20%) |
| Insomnia | 1 (20%) |
|  |  |
| **Ear and labyrinth disorders** |  |
| Any event | 1 (20%) |
| Ear pain | 1 (20%) |
|  |  |
| **Hepatobiliary disorders** |  |
| Any event | 1 (20%) |
| Jaundice | 1 (20%) |
|  |  |
| **Immune system disorders** |  |
| Any event | 1 (20%) |
| Graft versus host disease | 1 (20%) |
|  |  |
| **Injury, poisoning and procedural** |  |
| complications |  |
| Any event | 1 (20%) |
| Contusion | 1 (20%) |
|  |  |
| **Renal and urinary disorders** |  |
| Any event | 1 (20%) |
| Renal failure acute | 1 (20%) |

**Table S3 Summary of all Adverse Events, Part B**

| **System Organ Class Preferred Term** | **Placebo BID (N=20)** | **rhACE2 0.4 mg/kg BID (N=19)** |
| --- | --- | --- |
|  |  |  |
| ANY EVENT | 14 (70%) | 15 (79%) |
|  |  |  |
| **Metabolism and nutrition disorders** |  |  |
| Any event | 9 (45%) | 9 (47%) |
| Acidosis | 1 (5%) | 0 |
| Fluid overload | 0 | 1 (5%) |
| Food intolerance | 0 | 2 (11%) |
| Hyperglycaemia | 1 (5%) | 0 |
| Hyperkalaemia | 0 | 1 (5%) |
| Hypernatraemia | 0 | 4 (21%) |
| Hypochloraemia | 0 | 2 (11%) |
| Hypoglycaemia | 3 (15%) | 1 (5%) |
| Hypokalaemia | 5 (25%) | 6 (32%) |
| Hypomagnesaemia | 0 | 1 (5%) |
| Hypophosphataemia | 0 | 2 (11%) |
| Metabolic acidosis | 0 | 1 (5%) |
| Metabolic alkalosis | 0 | 1 (5%) |
|  |  |  |
| **Psychiatric disorders** |  |  |
| Any event | 7 (35%) | 9 (47%) |
| Agitation | 4 (20%) | 5 (26%) |
| Confusional state | 1 (5%) | 1 (5%) |
| Delirium | 3 (15%) | 4 (21%) |
| Hallucination | 1 (5%) | 1 (5%) |
| Insomnia | 2 (10%) | 3 (16%) |
| Suicidal ideation | 0 | 1 (5%) |
|  |  |  |
| **Gastrointestinal disorders** |  |  |
| Any event | 8 (40%) | 6 (32%) |
| Abdominal distension | 0 | 1 (5%) |
| Abdominal pain | 1 (5%) | 0 |
| Ascites | 1 (5%) | 0 |
| Constipation | 3 (15%) | 2 (11%) |
| Diarrhoea | 3 (15%) | 3 (16%) |
| Dysphagia | 1 (5%) | 5 (26%) |
| Gastrointestinal haemorrhage | 0 | 1 (5%) |
| Nausea | 1 (5%) | 2 (11%) |
| Toothache | 0 | 1 (5%) |
| Vomiting | 0 | 1 (5%) |
|  |  |  |
| **General disorders and administration site conditions** |  |  |
| Any event | 6 (30%) | 6 (32%) |
| Asthenia | 1 (5%) | 2 (11%) |
| Catheter site haemorrhage | 1 (5%) | 0 |
| Drug withdrawal syndrome | 0 | 1 (5%) |
| Face oedema | 0 | 1 (5%) |
| Generalised oedema | 3 (15%) | 3 (16%) |
| Local swelling | 0 | 1 (5%) |
| Multi-organ failure | 1 (5%) | 0 |
| Oedema peripheral | 0 | 1 (5%) |
| Pain | 1 (5%) | 3 (16%) |
| Peripheral swelling | 0 | 2 (11%) |
|  |  |  |
| **Respiratory, thoracic and mediastinal disorders** |  |  |
| Any event | 7 (35%) | 4 (21%) |
| Acute respiratory distress syndrome | 1 (5%) | 0 |
| Atelectasis | 0 | 1 (5%) |
| Epistaxis | 0 | 1 (5%) |
| Haemoptysis | 1 (5%) | 0 |
| Hiccups | 1 (5%) | 0 |
| Increased upper airway secretion | 0 | 1 (5%) |
| Laryngeal oedema | 0 | 1 (5%) |
| Pleural effusion | 4 (20%) | 0 |
| Pleuritic pain | 1 (5%) | 0 |
| Pneumothorax | 1 (5%) | 0 |
| Respiratory failure | 1 (5%) | 2 (11%) |
|  |  |  |
| **Blood and lymphatic system disorders** |  |  |
| Any event | 6 (30%) | 3 (16%) |
| Anaemia | 6 (30%) | 3 (16%) |
| Leukocytosis | 0 | 1 (5%) |
| Thrombocytopenia | 1 (5%) | 1 (5%) |
| Thrombocytosis | 0 | 2 (11%) |
|  |  |  |
| **Infections and infestations** |  |  |
| Any event | 2 (10%) | 7 (37%) |
| Candiduria | 0 | 1 (5%) |
| Clostridium difficile infection | 0 | 1 (5%) |
| Empyema | 1 (5%) | 0 |
| Fungal skin infection | 0 | 1 (5%) |
| Hepatitis C | 0 | 1 (5%) |
| Nasal herpes | 1 (5%) | 0 |
| Oral candidiasis | 1 (5%) | 1 (5%) |
| Oral herpes | 1 (5%) | 1 (5%) |
| Pneumonia | 0 | 3 (16%) |
| Pneumonia klebsiella | 0 | 1 (5%) |
| Pneumonia pseudomonal | 0 | 1 (5%) |
| Septic shock | 1 (5%) | 0 |
| Urinary tract infection | 0 | 1 (5%) |
|  |  |  |
| **Investigations** |  |  |
| Any event | 4 (20%) | 5 (26%) |
| Activated partial thromboplastin time prolonged | 0 | 1 (5%) |
| Alanine aminotransferase increased | 1 (5%) | 2 (11%) |
| Aspartate aminotransferase increased | 0 | 2 (11%) |
| Blood creatine phosphokinase increased | 0 | 1 (5%) |
| Blood lactate dehydrogenase increased | 1 (5%) | 0 |
| Blood pH increased | 1 (5%) | 0 |
| Blood potassium decreased | 0 | 1 (5%) |
| Blood pressure increased | 1 (5%) | 0 |
| Blood sodium increased | 0 | 1 (5%) |
| Electrocardiogram QT prolonged | 0 | 1 (5%) |
| Gamma-glutamyltransferase increased | 1 (5%) | 0 |
| Haemoglobin decreased | 1 (5%) | 1 (5%) |
| Hepatic enzyme increased | 1 (5%) | 0 |
| Platelet count increased | 0 | 1 (5%) |
| Respiratory rate increased | 1 (5%) | 0 |
| Urine output decreased | 1 (5%) | 0 |
| White blood cell count increased | 0 | 1 (5%) |
| **Skin and subcutaneous tissue disorders** |  |  |
| Any event | 1 (5%) | 8 (42%) |
| Blister | 0 | 1 (5%) |
| Blood blister | 0 | 1 (5%) |
| Erythema | 0 | 1 (5%) |
| Rash | 1 (5%) | 3 (16%) |
| Rash macular | 0 | 1 (5%) |
| Rash papular | 0 | 1 (5%) |
| Subcutaneous emphysema | 0 | 1 (5%) |
| Swelling face | 0 | 1 (5%) |
|  |  |  |
| **Nervous system disorders** |  |  |
| Any event | 3 (15%) | 3 (16%) |
| Dizziness | 1 (5%) | 0 |
| Encephalopathy | 0 | 2 (11%) |
| Headache | 2 (10%) | 2 (11%) |
|  |  |  |
| **Vascular disorders** |  |  |
| Any event | 4 (20%) | 2 (11%) |
| Deep vein thrombosis | 0 | 1 (5%) |
| Haematoma | 0 | 1 (5%) |
| Hypertension | 2 (10%) | 1 (5%) |
| Hypotension | 1 (5%) | 0 |
| Thrombophlebitis | 1 (5%) | 0 |
| Thrombophlebitis superficial | 0 | 1 (5%) |
|  |  |  |
| **Cardiac disorders** |  |  |
| Any event | 2 (10%) | 3 (16%) |
| Arrhythmia | 0 | 1 (5%) |
| Atrial fibrillation | 0 | 1 (5%) |
| Bradycardia | 0 | 1 (5%) |
| Cardiac arrest | 0 | 1 (5%) |
| Cyanosis | 0 | 1 (5%) |
| Pericardial effusion | 1 (5%) | 0 |
| Tachyarrhythmia | 0 | 1 (5%) |
| Ventricular tachycardia | 1 (5%) | 1 (5%) |
|  |  |  |
| **Injury, poisoning and procedural complications** |  |  |
| Any event | 3 (15%) | 1 (5%) |
| Bronchial anastomosis complication | 0 | 1 (5%) |
| Fall | 1 (5%) | 0 |
| Laceration | 1 (5%) | 0 |
| Penis injury | 1 (5%) | 0 |
| Wound | 1 (5%) | 0 |
|  |  |  |
| **Renal and urinary disorders** |  |  |
| Any event | 1 (5%) | 2 (11%) |
| Renal failure | 1 (5%) | 1 (5%) |
| Renal failure acute | 0 | 1 (5%) |
| **Hepatobiliary disorders** |  |  |
| Any event | 2 (10%) | 0 |
| Cholelithiasis | 1 (5%) | 0 |
| Hyperbilirubinaemia | 1 (5%) | 0 |
|  |  |  |
| **Musculoskeletal and connective tissue disorders** |  |  |
| Any event | 1 (5%) | 1 (5%) |
| Back pain | 1 (5%) | 1 (5%) |
|  |  |  |
| **Reproductive system and breast disorders** |  |  |
| Any event | 1 (5%) | 1 (5%) |
| Scrotal erythema | 0 | 1 (5%) |
| Scrotal oedema | 1 (5%) | 0 |
|  |  |  |
| **Ear and labyrinth disorders** |  |  |
| Any event | 1 (5%) | 0 |
| Ear pain | 1 (5%) | 0 |
|  |  |  |
| **Eye disorders** |  |  |
| Any event | 1 (5%) | 0 |
| Eye haemorrhage | 1 (5%) | 0 |
|  |  |  |
| **Surgical and medical procedures** |  |  |
| Any event | 0 | 1 (5%) |
| Endotracheal intubation | 0 | 1 (5%) |

Table S4 Summary of Drug-Related Adverse Events (Part B)

| **Preferred Term** | **Placebo BID**  **(n=20)** | **rhACE2 0.4 mg/kg BID**  **(n=19)** |
| --- | --- | --- |
| Any drug-related AEs, n (%) | 3 (15) | 3 (16) |
| Anemia | 1 (5) | 1 (5) |
| Diarrhoea | 0 | 2 (11) |
| Rash | 0 | 1 (5) |
| Rash macular | 0 | 1 (5) |
| Pericardial effusion | 1 (5) | 0 |
| Pneumonia | 0 | 1 (5) |
| Alanine aminotransferase increased | 1 (5) | 0 |
| Blood lactate dehydrogenase increased | 1 (5) | 0 |
| Gamma-glutamyltransferase increased | 1 (5) | 0 |
| Hypernatremia | 0 | 1 (5) |

**Table S5 Serious Adverse Events**

| **System Organ Class** | **Preferred Term** | **Part A** | **Part B** | |
| --- | --- | --- | --- | --- |
|  |  | **rhACE2 0.1 -> 0.2 -> 0.4 -> 0.8 mg/kg (n=5)** | **Placebo BID**  **(n=20)** | **rhACE2 0.4 mg/kg BID (n=19)** |
| Any event, n (%) | | 3 (60) | 4 (20) | 3 (16) |
| Infections and infestations | Influenza | 1 (20) | 0 | 0 |
|  | Sepsis | 1 (20) | 0 | 0 |
|  | Empyema | 0 | 1 (5) | 0 |
|  | Pneumonia | 0 | 0 | 1 (5) |
|  | Septic shock | 0 | 1 (5) | 0 |
| Respiratory, thoracic and mediastinal disorders | Acute respiratory distress syndrome | 1 (20) | 0 | 0 |
|  | Respiratory failure | 1 (20) | 0 | 0 |
|  | Pleural effusion | 0 | 1 (5) | 0 |
|  | Respiratory failure | 0 | 1 (5) | 1 (5) |
| Cardiac disorders | Asystole | 0 | 0 | 1 (5) |
| Blood and lymphatic system disorders | Disseminated intravascular coagulation | 1 (20) | 0 | 0 |
| Gastrointestinal disorders | Upper gastrointestinal hemorrhage | 1 (20) | 0 | 0 |
| General disorders and administration site conditions | Multi-organ failure | 1 (20) | 1 (5) | 0 |
| Immune system disorders | Graft versus host disease | 1 (20) | 0 | 0 |
| Injury, poisoning and procedural complications | Bronchial anastomosis complication |  | 0 | 1 (5) |
| Investigations | Hepatic enzyme increased |  | 1 (5) | 0 |
| Nervous system disorders | Subarachnoid hemorrhage | 1 (20) | 0 | 0 |
| Renal and urinary disorders | Renal failure acute | 1 (20) | 0 | 0 |

Table S6 Total SOFA Score and Change from Baseline (Part B)

| **Treatment** | **Planned Time** | **n** | **Mean (95% CI)** | |
| --- | --- | --- | --- | --- |
|  |  |  | **Total SOFA score** | **Change from Baseline** |
| Placebo BID (N=20) | Baseline | 20 | 7.8 (6.4, 9.1) | NA |
|  | Day 4 | 10 | 7.4 (5.2, 9.6) | -0.4 (-1.9, 1.1) |
|  | Follow-up Day 7 | 7 | 5.4 (2.7, 8.2) | -2.7 (-5.1, -0.3) |
| rhACE2 0.4 mg/kg BID (N=19) | Baseline | 18 | 8.9 (7.8, 10.1) | NA |
|  | Day 4 | 16 | 8.0 (6.3, 9.7) | -0.4 (-2.1, 1.3) |
|  | Follow-up Day 7 | 6 | 6.8 (4.1, 9.5) | -1.2 (-4.4, 2.0) |

**IMMUNOGENICITY**

Antibodies to GSK2586881 were measured using a validated electrochemiluminescence bridging assay. Testing was conducted using a tiered approach; samples were first tested in a screening assay, only samples found positive in this assay were tested in the confirmation assay.

There were 5 screen positive subjects identified during the study, 1 in Part A and 4 in the placebo group of Part B. None of these samples was positive in the confirmation assay.

**PHARMACOKINETICS**

**Assay methods**Concentrations of GSK2586881 were measured in plasma using a validated sandwich chemiluminescent immunoassay using commercial antibodies (R&D Systems, Abingdon, UK and Sigma-Aldrich Poole, UK) followed by a horse-radish peroxidase (HRP) labelled goat anti-rabbit IgG (Thermo Fisher Scientific, Hemel Hempstead, UK). The assay detects native human soluble ACE2 and recombinant human ACE2 (GSK2586881).

**Pharmacokinetic Modeling**GSK21586881 plasma concentration-time data were analyzed using the non-linear mixed effects modeling approach implemented in NONMEM software (version VII, release 7.2; Icon Development Solutions, Ellicott City, Maryland, USA), Intel Fortran 12.1.4 and executed on a Unix grid. The results were analyzed using the statistical software package R (version 2.1.3.1).Parameters were estimated using the first order conditional estimation method with interaction between the two levels of stochastic effects (FOCE interaction, METHOD=1) and ADVAN 6.

Utilizing NONMEM standard coding PK parameters for each individual (Pi) were modelled according to equation 1.

** (1)**

Where θ is the population estimate for the parameter and η_i_ is the random deviation of Pi from the population estimate (P). The values of η_i_ are assumed normally distributed with a mean of zero and a variance of ω^2^**.** Residual error was described using a log normal distribution via an additive residual error model on the log transformed values as described in equation 2.

 (2)

Where C_mij_ represents the jth natural log transformed concentration from the ith individual and C_pij_ represents the predicted concentration or EEG measurement on a normal scale. The values of ε are assumed normally distributed with a mean of zero and a variance of σ ^2^.

Goodness-of-fit was determined using; the minimum value of the objective function (defined as minus twice the log-likelihood), visual inspection of the plots of predictions, diagnostic plots of conditional weighted residuals and parameter relative standard error (%). To determine if nested models were statistically significantly different the change in the objective function (OBJ) and number of free parameters (degrees of freedom or d.f.) were compared against a Chi-squared table. Final models were additionally examined by running visual predictive checks (VPC) to compare the predicted range to the observations.

**Results**GSK2586881 was quantifiable (≥100 ng/mL) in the plasma of all ARDS subjects from whom a PK sample was collected and analysed up to 12 hours post-dose (Part B) (Figure E1). A one-compartmental linear model with 1^st^ order elimination accounting for some inter-individual variability in clearance was found to adequately describe GSK2586881 PK data (i.e. concentration profiles over time). The effective plasma half-life was estimated to be between 6-10 hours, and GSK2586881 PK was similar to data generated in healthy subjects (Haschke M, , et al. *Clin Pharmacokinet* 52:783, 2013). The volume of distribution for GSK2586881 was estimated to be 5.8 L (population estimate).

**Figure S3**: Plasma concentration-time plots (linear [A, C] and semi-log [B, D]) for GSK2586881. (A, B) Part A, doses 0.1, 0.2, 0.4 and 0.8 mg/kg; (C, D) Part B, 0.4 mg/kg twice daily

**Figure S4**. Scatterplot of GSK2586881 Plasma Concentration vs ACE2 Enzymatic activity. LLQ for PK conc. = 100 ng/mL; LLQ for ACE2 Enzyme activity. = 390 ng/mL

References

1. National Heart, Lung, and Blood Institute Acute Respiratory Distress Syndrome (ARDS) Clinical Trials Network, Wheeler AP, Bernard GR, Thompson BT, Schoenfeld D, Wiedemann HP, deBoisblanc B, Connors AF Jr, Hite RD, Harabin AL. Pulmonary-Artery versus Central Venous Catheter to Guide Treatment of Acute Lung Injury. *N Engl J Med.* 2006; 25:354(21): 2213-24.

2. National Heart, Lung, and Blood Institute Acute Respiratory Distress Syndrome (ARDS) Clinical Trials Network, Wiedemann HP, Wheeler AP, Bernard GR, Thompson BT, Hayden D, deBoisblanc B, Connors AF Jr, Hite RD, Harabin AL. Comparison of Two Fluid-Management Strategies in Acute Lung Injury. *N Engl J Med.* 2006; 354(24): 2564-75.
