# Supplementary material for: A pilot clinical trial of recombinant human angiotensin-converting enzyme 2 in acute respiratory distress syndrome
Source: Crit Care. 2017 Sep 7;21:234. doi: 10.1186/s13054-017-1823-x (PMC5588692; doi:10.1186/s13054-017-1823-x)
Supplement: Supplementary file 2 — Ethics Committees and Institutional Review Boards. (DOCX 13 kb) [file 13054_2017_1823_MOESM2_ESM.docx]

**CRIC-D-17-00258:** Khan et al, “A Pilot Clinical Trial of Recombinant Human Angiotensin Converting Enzyme 2 in Acute Respiratory Distress Syndrome”

**Ethics Committees/IRBs**

Boyd: UBC-Providence Health Care Research Ethics Board, 1081 Burrard Street, 11th Floor Hornby Site – SPH, Vancouver, British Columbia, V6Z 1Y6

Hall: Capital Health REB, 5790 University Ave Room 118, Halifax, Nova Scotia, B3H 1V7

Lellouche: Comité d'éthique de la recherche de l'Institut universitaire de cardiologie et de pneumologie de Québec, 2725 Chemin Ste-Foy, Québec G1V 4G5

Poirier: Hopital Charles LeMoyne, 3120 boul. Taschereau, Porte E-302, Greenfield Park, Québec, J4V 2H1

Ronco: University of British Columbia, 828 West 10th Avenue, Room 210, Vancouver, British Columbia, V5Z 1L8

Christie: University of Pennsylvania, Office of Regulatory Affairs, 3624 Market St., Suite 301 S, Philadelphia, PA 19104-6006

Khan: Oregon Health & Science University, Research Integrity Office, L106-RI, 3181 SW Sam Jackson Park Road, Portland, OR 97239-3098

Tidswell: Baystate Medical Center Institutional Review Board, 759 Chestnut Street, Springfield MA 01199

Zeno: Western Institutional Review Board, 1019 39th Avenue SE Suite 120, Puyallup, WA 98374-2115

Albertson: University of California Davis, Office of Research, IRB Administration, 1850 Research Park Drive, Suite 300, Davis, CA, 95618-6153
